# Differential Acute Effects of Selenomethionine and Sodium Selenite on the Severity of Colitis

**DOI:** 10.3390/nu7042687

**Published:** 2015-04-10

**Authors:** Franziska Hiller, Lisa Oldorff, Karolin Besselt, Anna Patricia Kipp

**Affiliations:** German Institute of Human Nutrition Potsdam-Rehbruecke (DIfE), Arthur-Scheunert-Allee 114-116, 14558 Nuthetal, Germany; E-Mails: franziska.hiller@dife.de (F.H.); lisa.oldorff@gmx.de (L.O.); karolin.besselt@dife.de (K.B.)

**Keywords:** selenomethionine, selenite, colitis, IBD, DSS, oxidative stress

## Abstract

The European population is only suboptimally supplied with the essential trace element selenium. Such a selenium status is supposed to worsen colitis while colitis-suppressive effects were observed with adequate or supplemented amounts of both organic selenomethionine (SeMet) and inorganic sodium selenite. In order to better understand the effect of these selenocompounds on colitis development we examined colonic phenotypes of mice fed supplemented diets before the onset of colitis or during the acute phase. Colitis was induced by treating mice with 1% dextran sulfate sodium (DSS) for seven days. The selenium-enriched diets were either provided directly after weaning (long-term) or were given to mice with a suboptimal selenium status after DSS withdrawal (short-term). While long-term selenium supplementation had no effect on colitis development, short-term selenite supplementation, however, resulted in a more severe colitis. Colonic selenoprotein expression was maximized in all selenium-supplemented groups independent of the selenocompound or intervention time. This indicates that the short-term selenite effect appears to be independent from colonic selenoprotein expression. In conclusion, a selenite supplementation during acute colitis has no health benefits but may even aggravate the course of disease.

## 1. Introduction

Inflammatory bowel disease (IBD) is characterized by a relapsing inflammation of the gastro-intestinal tract. The two main types of IBD are Crohn’s disease (CD) and ulcerative colitis (UC). The prevalence and incidence of IBD raise especially in the industrialized countries, but the precise etiology of the disease is still unknown [1]. Clearly, nutrition has an impact on disease development. Many IBD patients suffer from general malnutrition, but also specific micronutrients are less well absorbed [2]. One of those micronutrients is the essential trace element selenium, which is reduced systemically in many CD patients [3].

The main functions of selenium are mediated by selenoproteins in which it is incorporated as selenocysteine (Sec) [4]. These selenoproteins can be found in all tissues, but in varying amounts [5]. In immune cells, the glutathione peroxidases (GPx) 1 and 4, as well as selenoproteins S and K are best characterized [6]. GPx1 and 4 are involved in the activation of phagocytes by protecting them against H_2_O_2_ and lipid peroxidation [7,8]. Additionally, GPx1 has been shown to play a role in T helper (Th) cell differentiation during allergen-induced airway inflammation [9]. SelK is necessary for immune cell activation by promoting Ca^2+^ flux [10], while SelS regulates cytokine production in macrophages [11]. Thus, selenium and the entailed selenoprotein deficiency will result in compromised immune responses [12,13,14,15]. For instance, microarray analyses identified lower expression levels of genes of the inflammatory response in splenic leukocytes with a suboptimal selenium status [12]. As selenium supply is suboptimal in Europe [16], especially individuals suffering from chronic immune diseases, such as IBD, may consider selenium supplementation to optimize their immune response. In the human diet, selenium is mainly provided as the organic seleno-amino acid selenomethionine (SeMet) [16]. However, many feeding trials in mice have been performed with inorganic sodium selenite [17,18].

**Figure 1:**
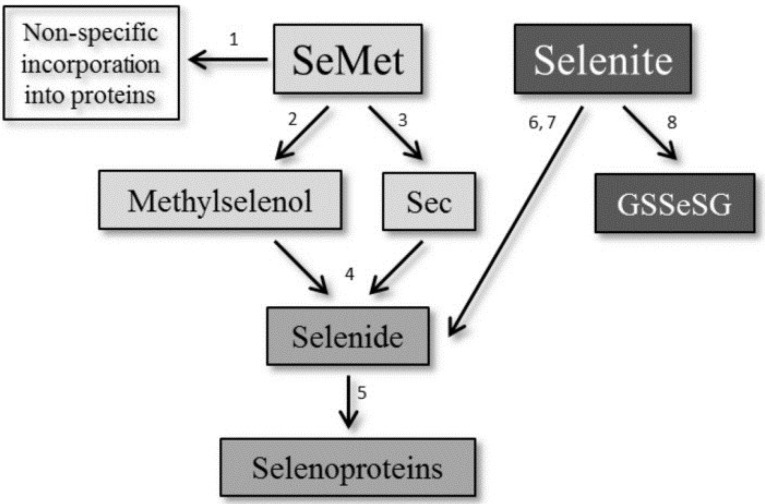
Metabolism of selenomethionine (SeMet) and selenite. (1) SeMet can be incorporated non-specifically into proteins instead of methionine [22]. Otherwise, it is transformed enzymatically either by (2) γ-lyase into methylselenol [23] or through (3) transselenation into selenocysteine (Sec) [24]. (4) Both are further degraded to selenide, which is used to (5) newly synthetize Sec directly at the tRNA^Ser/Sec^. Selenite is either metabolized by the (6) glutaredoxin [25] or by the (7) thioredoxin [26] system yielding selenide. (8) Alternatively, selenite may react with intracellular glutathione (GSH) to form selenodiglutathione (GSSeSG) [27].

Both selenocompounds are absorbed and metabolized differently in the body (Figure 1). SeMet is actively transported through the intestinal mucosa, resulting in highly effective absorption [19], while selenite is absorbed passively [20]. Based on these differences, the question arises whether SeMet and selenite have advantages over each other in improving the selenium status and, thus, colitis severity. In independent studies using a colitis model induced by dextran sulfate sodium (DSS), selenium-deficient mice were either compared to SeMet- or selenite-fed mice. Both selenocompounds reduced the severity of colitis in comparison to selenium deficiency [17,18,21]. Therefore, we directly compared diets supplemented with SeMet or sodium selenite during DSS-induced colitis. Additionally, we also investigated putative differences between a long-term supplementation starting before the onset of the colitis and a short-term supplementation starting during the acute inflammation. Surprisingly, none of the long-term supplementations had an effect on colitis development compared to a suboptimal selenium supply in this moderate DSS model. However, supplementation with selenite during the acute inflammation even exacerbated the colitis.

## 2. Experimental Section

### 2.1. Animal Experiment

C57BL/6J mice were housed individually in independently ventilated cages under specific-pathogen-free conditions with a 12-h light/dark cycle and free access to food and water. Mice were fed a Torula yeast-based chow (Altromin C1045, Lage, Germany), which had a basal selenium content of 0.07 mg/kg.

For the selenium-supplemented diets, the basal chow was enriched either with l(+)-selenomethionine (Fisher Scientific, Schwerte, Germany) or with sodium selenite (Sigma Aldrich, Steinheim, Germany) to a final selenium content of 0.6 mg/kg, which is about four-times the adequate intake of mice [28]. The selenium concentration was measured fluorometrically [29].

**Figure 2:**
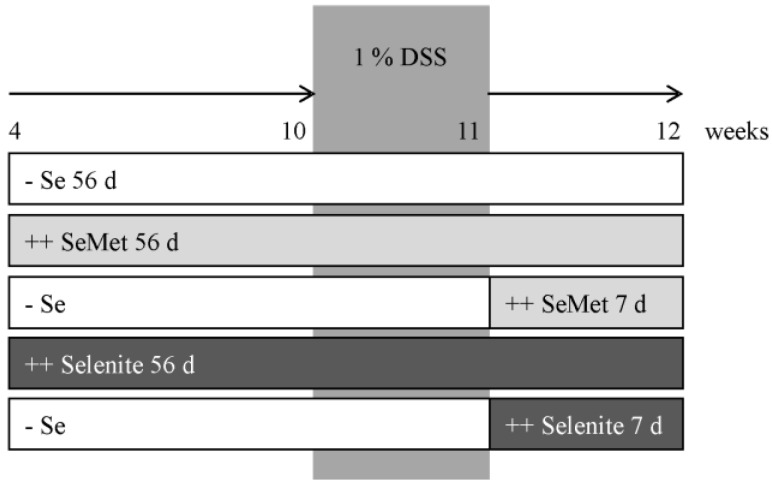
Study design. At the age of 4 weeks, mice were adjusted either to the suboptimal selenium diet (− Se) or to diets supplemented with selenomethionine (SeMet) or selenite (++ SeMet 56 days or ++ Selenite 56 day). At the age of 10 weeks, they received 1% DSS via the drinking water for one week. With DSS withdrawal, short-term supplementation of mice with a suboptimal selenium status started for another week (++ SeMet 7 days, ++ Selenite 7 days).

At the age of four weeks, mice were adjusted either to a supplemented or suboptimal selenium diet for six weeks. Afterwards, mice received 1% DSS (w/v, 36–50 kDa, MP Biomedicals, Illkirch, France) via the drinking water (sterile-filtered) *ad libitum* for seven days. With DSS withdrawal, the short-term supplementation with either SeMet or sodium selenite started for another week (Figure 2). Then mice were anesthetized with isoflurane (Abbot, Wiesbaden, Germany) and blood was withdrawn by heart puncture into heparinized tubes. Plasma was obtained after centrifugation of the blood for 15 min (1200 × g, 4 °C). Anesthetized animals were sacrificed by cervical dislocation. Tissue sampling was performed as reported [21].

Animal experiments were performed in compliance with the German animal protection law (Tierschutzgesetz). The mice were housed and handled in accordance with good animal practice as defined by the Federation of Laboratory Animal Science Associations (FELASA, www.felasa.eu/) and the national animal welfare body (GV-SOLAS, www.gv-solas.de/). The animal welfare committees of the German Institute of human Nutrition (DIfE) as well as the local authorities (Landesamt für Umwelt, Gesundheit und Verbraucherschutz, Brandenburg, Germany) approved all animal experiments (project number V3-2347-29-2012, date of approval: 26 October 2012).

### 2.2. Scoring Systems

The inflammation score was based on clinical, macro- and microscopic parameters according to earlier publications with some modifications [21,30]. From the beginning of DSS treatment until the end of the experiment, weight, diarrhea and blood in the feces were monitored. Weight loss at the end of the study was calculated as the percentage of the maximal weight during the monitoring phase and scored as follows: <5% = 0, 5%–10% = 1, 11%–15% = 2, 16%–20% = 3 and >20% = 4. Diarrhea was further categorized into abnormally-shaped feces (1 point (pt)), soft and slimy feces (2 pts) and watery feces (3 pts). Diarrhea-points were summed and scaled-down as follows: 0 pts = 0, 1–10 pts = 1, 11–20 pts = 2, >20 pts = 3. The number of days with visible blood in the feces were added and scored: none = 0, 1–3 days = 1, 4–6 days = 2 and >6 days = 3. Colon length was measured from caecum to anus (<7.5 cm = 1 pt). Swelling of the colon was evaluated as low-grade (1 pt), moderate (2 pts) or severe swelling (3 pts). Hematoxylin and eosin (H & E)-stained colon Swiss rolls (see: *2.3. immunohistochemistry and histochemistry*) were examined and scored for edema of the mucosa/submucosa, hemorrhage, loss of mucosa, disturbed crypt architecture and inflammatory infiltrate, as described before [21,30]. Finally, the total inflammation score was summarized.

8-Hydroxy-2’-deoxyguanosin (8-OHdG) staining intensity of epithelial cells was also carried out in colon Swiss rolls (see *2.3. immunohistochemistry and histochemistry*). Every intact crypt was evaluated according to its staining intensity: no (0 pt), light (1 pt) or intense (2 pts) staining. The mean crypt staining intensity per mouse was calculated.

### 2.3. Immunohistochemistry and Histochemistry

Swiss rolls were made from colon tissues (transverse part to anus). Therefore, the colon was washed with 4% formaldehyde (Roth, Karlsruhe, Germany), opened longitudinally, coiled up and fixed in 4% formaldehyde. Colon rolls were embedded in paraffin, and serial sections (2 μm) were prepared for H & E and immunohistochemical (IHC) staining [31,32]. IHC was performed with mouse 8-OHdG antibody (ab26842, Abcam, Cambridge, UK) diluted 1:200, rabbit SelH antibody (ab151023, Abcam, Cambridge, UK) diluted 1:300 and rat F4/80 antibody (MCA497, AbD Serotec, Kidlington, Oxford, UK) diluted 1:8000. N-Histofine^®^ Simple Stain Mouse MAX PO (R) anti-rabbit, N-Histofine^®^ Simple Stain MAX PO (M) anti-mouse or N-Histofine^®^ Simple Stain Mouse MAX PO (R) anti-rat (all from Nichirei Biosciences, Tokyo, Japan) were used as secondary antibodies.

### 2.4. Enzyme Activities

Samples were prepared as described before [33]. Measurement of NQO1 [34], TrxR [21] and GPx [33] activities were also described previously. Briefly, NQO1 activity was examined by menadione-mediated reduction of 3-(4, 5-dimethylthiazol-2-yl)-2, 5-diphenyltetrazolium bromide (MTT). TrxR activity was measured by the nicotinamide adenine dinucleotide phosphate (NADPH)-dependent reduction of 5, 5’-dithiobis(2-nitrobenzoic acid) (DTNB). GPx activity was determined in a NADPH-consuming glutathione reductase coupled assay. All measurements were carried out in 96-well format using a microplate reader (Synergy2, BioTek, Bad Friedrichshall, Germany).

### 2.5. Western Blotting

Protein analysis for Sepp1 was carried out with 1 μL of plasma per animal. SDS-polyacrylamide gel electrophoresis and Western blotting were performed as described previously [35]. Sepp1 was detected by anti-Sepp1 antibody (1:500, kindly provided by Prof. Schomburg, Charité Berlin). Horseradish peroxidase-conjugated goat anti-rabbit antibody (Rockland, Hamburg, Germany) served as the secondary antibody. The intensity of identified bands was quantified densitometrically with the Luminescent Image Analyser LAS-3000 system (Fujifilm, Tokyo, Japan). Protein expression was normalized to Coomassie blue gel staining.

### 2.6. Cytokine Analysis

In plasma, concentrations of TNFα, IFNγ, IL-4, IL-5, IL-6 and IL-12p70 were measured with the ProcartaPlex Mouse Essential Th1/Th2 Cytokine Panel (eBioscience, Frankfurt/Main, Germany), according to the manufacturer’s protocol. Prior to application, plasma samples were centrifuged with 10,000 x g for 10 min at 4 °C. Incubation with samples took place over night at 4 °C. Luminescence was detected by the Bio-Plex 200 system (Bio-Rad Laboratories, München, Germany).

### 2.7. Quantitative Real-Time PCR

RNA analysis was carried out in samples of the proximal colon thatwere snap-frozen and pulverized with the TissueLyser (Qiagen, Hilden, Germany). The mRNA was extracted with the Dynabeads^®^ mRNA DIRECT™ Kit (Ambion, Darmstadt, Germany), according to the manufacturer’s protocol. 150 ng of mRNA from colon tissue was transcribed into cDNA by reverse transcriptase PCR using 150 fmol oligo (dT) 15 primers and 180 U Moloney Murine Leukemia Virus Reverse Transcriptase (M-MLV RT, Promega, Mannheim, Germany). Real-time PCR was performed with 1 μL of 1:2 diluted cDNA, as described before [36]. The sequences of the primers are listed in Table 1. Data were normalized to non-selenium-responsive Rpl13a.

**Table 1:** Primer sequences (5’→3’).

Gene	Sequence		Species
**Cox-2**	fwd	TTCAAGACAGATCATAAGCGAG	mouse/human
	rev	GTGGCATACATCATCAGACCA	
**Gpx1**	fwd	CACACCAGGAGAATGGCAAGA	mouse
	rev	GAACTTCTCAAAGTTCCAGGCA	
**Ifnγ**	fwd	GCCAAGTTTGAGGTCAACAACCC	mouse
	rev	CCGAATCAGCAGCGACTCCT	
**Rpl13a**	fwd	GTTCGGCTGAAGCCTACCAG	mouse
	rev	TTCCGTAACCTCAAGATCTGCT	
**Selh**	fwd	CCTTATTCCACCAACGCGCCA	mouse
	rev	GCGTCAGCTCGTACAATGCTC	
**Sepw1**	fwd	ATGCCTGGACATTTGTGGCGA	mouse
	rev	GCAGCTTTGATGGCGGTCAC	
**Sepp1**	fwd	CTCATCTATGACAGATGTGGCCGT	mouse
	rev	AAGACTCGTGAGATTGCAGTTTCC	
**Tnfα**	fwd	CCACGTCGTAGCAAACCACC	mouse
	rev	TACAACCCATCGGCTGGCAC	

### 2.8. Statistical Analysis

**Figure 3:**
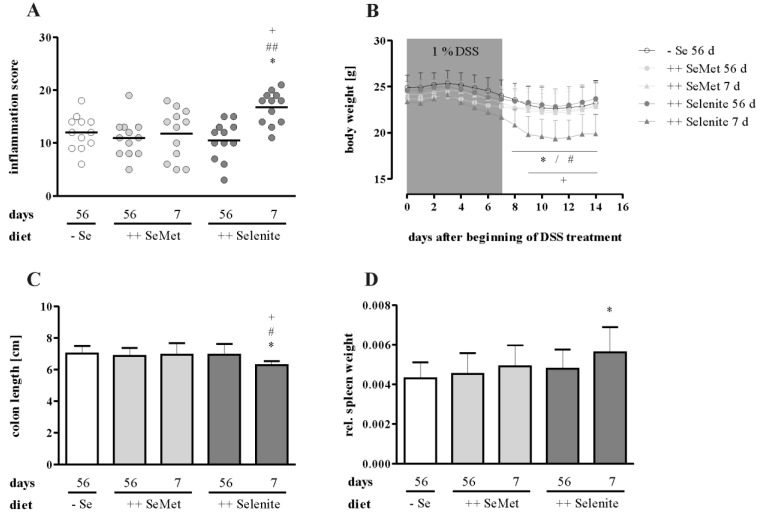
Intestinal inflammation is enhanced by short-term selenite supplementation. (**A**) The severity of inflammation was assessed by a score that was based on clinical, macroscopic and microscopic parameters. Each point represents one mouse. Lines indicate means. Significant differences were calculated by one-way ANOVA and Bonferroni’s posttest. (**B**) Body weight was monitored daily from the beginning of the DSS treatment until the end of the experiment. Significant differences were calculated by two-way ANOVA and Bonferroni’s posttest. (**C**) Colon length was measured from caecum to anus. Significant differences were calculated by one-way ANOVA and Bonferroni’s posttest. (**D**) Spleen weight was normalized to the body weight. Data are shown as the mean + SD. *n* = 12. Significant differences were calculated by one-way ANOVA and Bonferroni’s posttest. * *p* < 0.05 *vs.* − Se; ^#^ *p* < 0.05, ^##^ *p* < 0.01 *vs.* ++ Selenite 56 days; ^+^ *p* < 0.05 *vs.* ++ SeMet 7 days.

**Figure 4:**
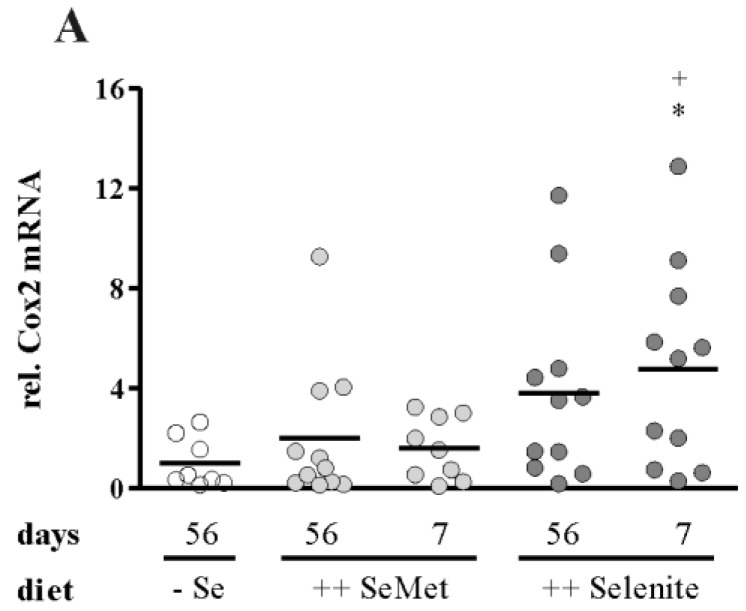

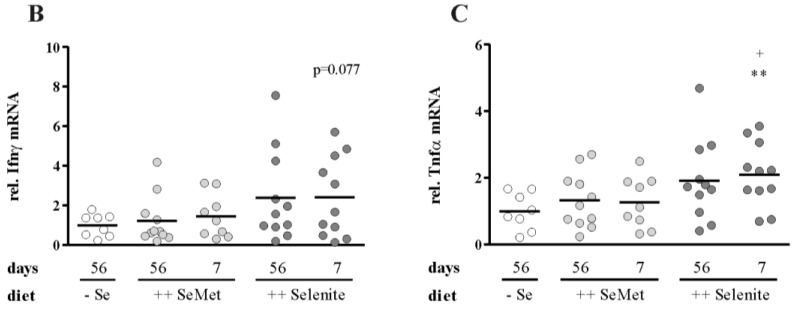
Transcript levels of pro-inflammatory markers are upregulated by short-term selenite supplementation. Colonic mRNA expression of (**A**) Cox2, (**B**) Ifnγ and (**C**) Tnfα was measured by qPCR and normalized to Rpl13a. Each point represents one mouse. Lines indicate means. Significant differences were calculated by Student’s *t*-test. * *p* < 0.05, ** *p* < 0.01, *p* = 0.077 *vs*. − Se; ^+^ *p* < 0.05 *vs*. ++ SeMet 7 days.

**Figure 5:**
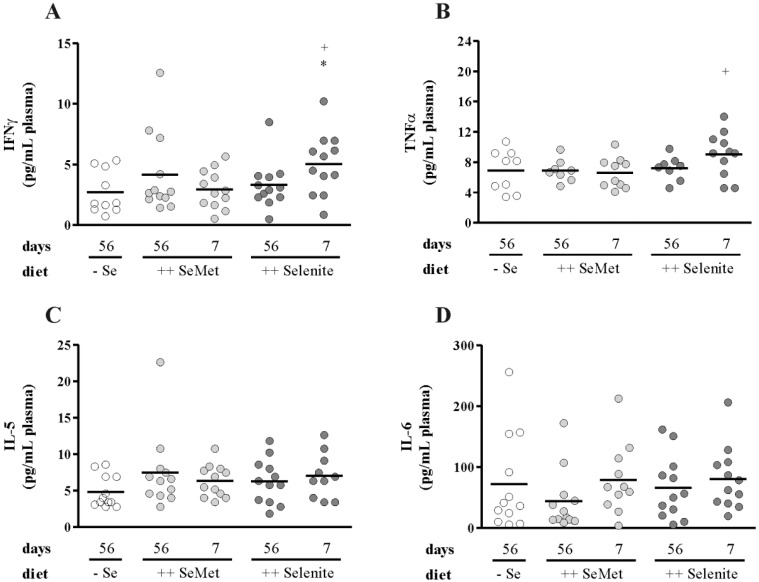
Th1-cytokines are increased in plasma of short-term selenite supplemented mice, while Th2-cytokines are unaffected. (**A**) IFNγ, (**B**) TNFα, (**C**) IL-5 and (**D**) IL-6 plasma levels were measured by multiplex immunoassay. Each point represents one mouse. Lines indicate means. Significant differences were calculated by Student’s *t*-test. * *p* < 0.05 *vs*. − Se 56 days; ^+^ *p* < 0.05 *vs*. ++ SeMet 7 days.

Data are shown as the mean + SD (standard deviation). The normal distribution of the data was tested with the Shapiro-Wilk’s Test. Statistical significance was calculated by GraphPad Prism Version 6 (San Diego, CA, US). We used one-way ANOVA with Bonferroni’s posttest as the standard test. Only for the body weight loss (Figure 3B) a two-way ANOVA with Bonferroni’s posttest was performed. In Figure 4 and Figure 5, the results of an unpaired Student’s *t*-test are shown, because the one-way ANOVA did not result in significant differences. Further information is provided by the figure legends. A *p*-value < 0.05 was considered as statistically significant.

## 3. Results

### 3.1. Selenium Status at Colitis Onset Did not Influence Inflammation Severity

Prior to colitis induction, mice were fed either a suboptimal selenium diet (− Se 56 days) or diets enriched with four-fold the amount of the recommended intake of selenium for mice [28]. Enriched diets contained selenium either as SeMet (++ SeMet 56 days) or selenite (++ Selenite 56 days). To induce a non-lethal colitis, mice were treated with 1% DSS (w/v) for seven days. One week after DSS withdrawal, the overall inflammation score did not differ between the three long-term (56 d) supplementation groups (Figure 3A). Accordingly, further markers of inflammation, such as body weight loss (Figure 3B), colon length (Figure 3C) and relative spleen weight (Figure 3D), were not altered. Furthermore, no differences were observed for colonic mRNA expression of Cox2 (Figure 4A), Ifnγ (Figure 4B), or Tnfα (Figure 4C) nor for plasma cytokine levels of IFNγ (Figure 5A), TNFα (Figure 5B), IL-5 (Figure 5C) or IL-6 (Figure 5D). Thus, optimizing the selenium status before the onset of colitis did not attenuate inflammation.

### 3.2. Selenite Supplementation during Acute Inflammation Increased Parameters of Colitis

To investigate if the time point of selenium intervention is of relevance, we additionally examined short-term supplementations during acute colitis. Mice with a suboptimal selenium status were fed either SeMet- (++ SeMet 7 days) or selenite- (++ Selenite 7 days) enriched diets starting from DSS withdrawal for another week. The assessment of colitis severity revealed an enhanced inflammation score in the short-term selenite supplemented mice compared to all other groups, while short-term SeMet treatment had no effect (Figure 3A). The body weight of short-term selenite supplemented mice was significantly lower than that of − Se and long-term selenite supplemented mice from Day 8, as well as of short-term SeMet from Day 9 until the end of the experiment (Figure 3B). The colon of the ++ Selenite 7 days mice was shorter (Figure 3C), and their relative spleen weight was increased (Figure 3D), also indicating a more severe inflammation. Colonic mRNA expression of the key enzyme of prostaglandin synthesis Cox2 (Figure 4A) and the Th1-cytokines Ifnγ (Figure 4B) and Tnfα (Figure 4C) was elevated. Correspondingly, plasma levels of IFNγ (Figure 5A) and TNFα (Figure 5B) were significantly increased by short-term selenite supplementation compared to the short-term SeMet and the suboptimal selenium group or to the short-term SeMet group only. These concentrations were measured with a 6-Plex cytokine panel along with IL-4, IL-5, IL-6 and IL-12. While IL-4 and IL-12 were below the limits of detection, the Th2-cytokines IL-5 (Figure 5C) and IL-6 (Figure 5D) were not altered by any dietary intervention. These results point towards an enhancement of Th1-driven DSS-colitis by selenite supplementation during acute inflammation.

### 3.3. Enhancement of Colitis by Short-Term Selenite Supplementation Did Not Correlate with Changes in Selenoprotein Expression

**Figure 6:**
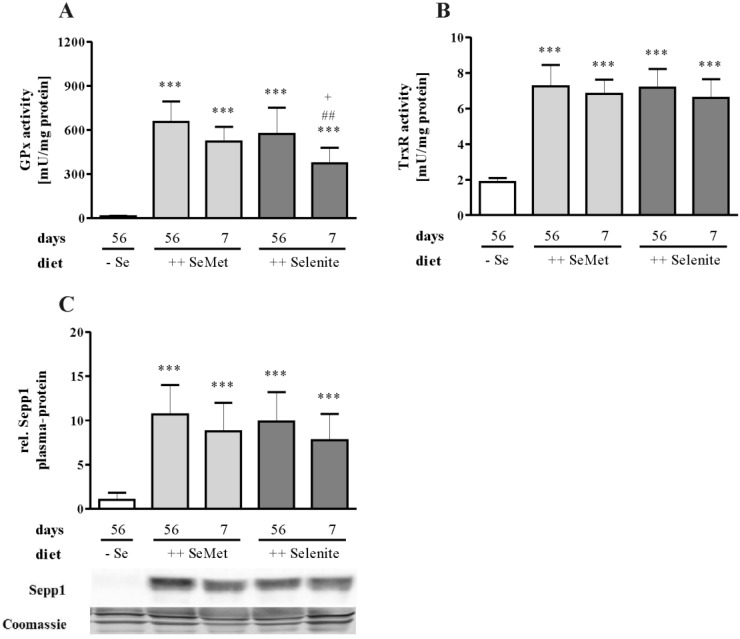
Systemic selenium status is not replete after short-term supplementation with selenite. Total (**A**) GPx and (**B**) TrxR activity was measured in homogenates of the liver. (**C**) Sepp1 protein levels in the plasma were detected by Western Blot analysis and normalized to Coomassie blue gel staining. Representative bands for each intervention group are shown. Data are depicted as the mean + SD. *n* = 12. Significant differences were calculated by one-way ANOVA and Bonferroni’s posttest. ** *p* < 0.01, *** *p* < 0.001 *vs*. − Se; ^##^ *p* < 0.01 *vs*. ++ Selenite 56 days; ^+^ *p* < 0.05 *vs*. ++ SeMet 7 days.

To check the repletion of the selenium status, selenoenzyme activities, as well as mRNA expression of selenoproteins were measured. All analyzed parameters were significantly downregulated in the suboptimal selenium control group. In the liver, the central organ of whole body selenium supply [37], total GPx activity did not reach the same level after short-term selenite supplementation compared to long-term supplementation and short-term SeMet supplementation (Figure 6A). Therefore, the systemic selenium status was not fully replete after one week of selenite supplementation. Furthermore, short-term selenite supplementation was not as effective at optimizing hepatic selenium status as the respective SeMet treatment. In contrast, hepatic TrxR activity was already replete after short-term selenite supplementation (Figure 6B). The same applies to Sepp1 in the plasma, another well-established marker of systemic selenium status (Figure 6C) [38].

**Figure 7:**
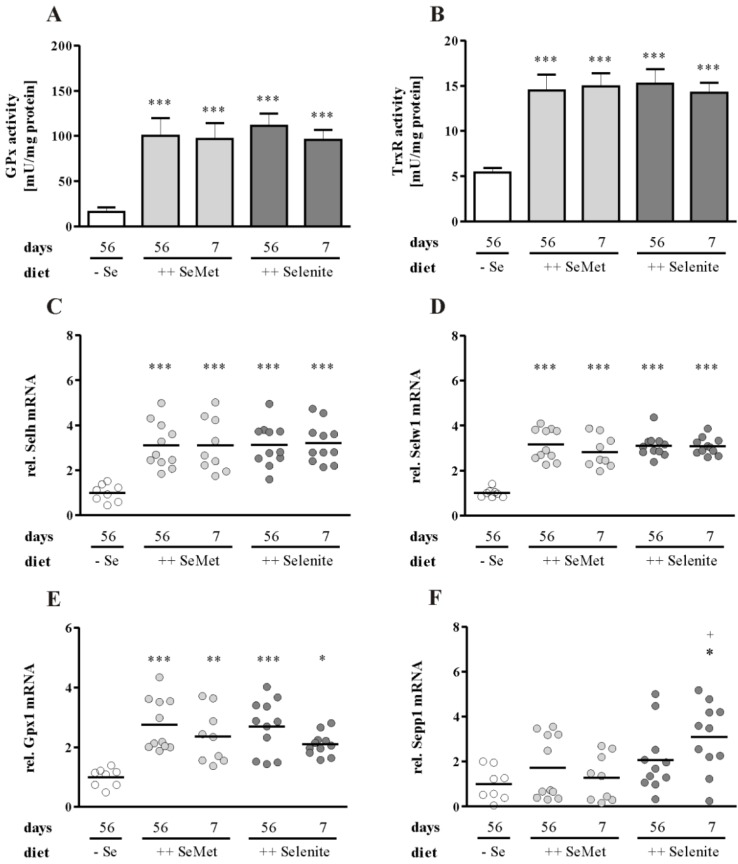

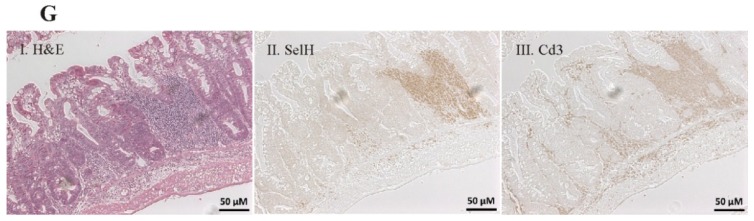
Colonic selenium status is replete after short-term supplementation with SeMet and selenite. Total (**A**) GPx and (**B**) TrxR activity was measured in homogenates of the colon. Data are shown as the mean + SD. *n* = 12. Colonic mRNA expression of (**C**) Selh, (**D**) Selw1, (**E**) Gpx1 and (**F**) Sepp1 was measured by qPCR and normalized to Rpl13a. Each point represents one mouse. Lines indicate means. Significant differences were calculated by one-way ANOVA and Bonferroni’s posttest. * *p* < 0.05, ** *p* < 0.01, *** *p* < 0.001 vs. − Se; ^+^ *p* < 0.05 vs. ++ SeMet 7 days. (**G**) Colon Swiss rolls were stained with: I. H & E, II. SelH antibody, III. CD3 antibody. Representative pictures are shown for mice with suboptimal selenium supply (− Se) depicting inflamed tissue.

In the colon, short-term supplementation increased the GPx activity to the same level as the long-term supplementation independent of the selenocompound (Figure 7A). The same was observed for TrxR activity (Figure 7B), as well as for mRNA expression of the selenium-sensitive selenoproteins Selh (Figure 7C), Selw1 (Figure 7D) and Gpx1 (Figure 7E). Thus, mice appear to be close to an optimized selenium status after seven days of supplementation, because only hepatic GPx activity was not fully saturated yet. In the colon, selenoprotein expression and activity was replete after one week of supplementation with both SeMet and selenite. This indicates that the pro-inflammatory effect of short-term selenite appears to be independent of the changes in selenoprotein expression. One exception was observed for the transcript levels of colonic Sepp1, which were upregulated in the short-term selenite supplemented group (Figure 7F). In intraepithelial T-cells, SelH protein expression was unexpectedly still detectable under suboptimal selenium supply (Figure 7G).

### 3.4. The Cellular Redox Status Is Unaffected after Seven Days of Selenite Supplementation

**Figure 8:**
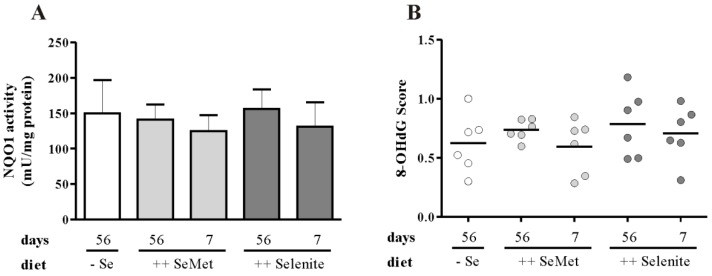

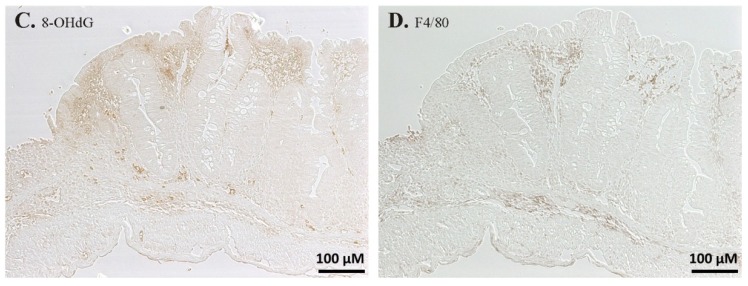
Markers of oxidative stress are unaffected by selenite feeding. (**A**) NQO1 activity was measured in homogenates of the colon. Data are shown as the mean + SD. *n* = 12. (**B**) 8-OHdG staining intensity of epithelial cells was scored in six representative animals per group. Each point represents one mouse. Lines indicate means. Colon Swiss rolls were stained immunohistochemically with (**C**) anti-8-OHdG antibody and (**D**) anti-F4/80 antibody.

To further elucidate putative mechanisms for the pro-inflammatory effects of short-term selenite supplementation, we analyzed different markers of the cellular redox status, because selenite is known to act pro-oxidatively [39,40,41]. The redox-regulated Nrf2 target gene NQO1 (Figure 8A), however, was unaffected by the short-term selenite treatment. Furthermore, oxidative DNA damage was evaluated by IHC staining of 8-OHdG in colon tissue. In epithelial cells of normal appearing crypts, no difference between the selenium diets was observed after scoring the staining intensities (Figure 8B). 8-OHdG levels were higher in inflammatory cells (Figure 8C), which are mainly supposed to be F4/80-positive macrophages (Figure 8D). Thus, the level of oxidative stress in epithelial cells appeared to be similar in all experimental groups at the time point of examination.

## 4. Discussion

In this study, we could not reproduce earlier results of different groups, which identified anti-inflammatory effects of SeMet and selenite during colitis. In previous studies, selenium deficiency caused a more severe inflammation than an adequate or supplemented supply. The experimental conditions used, though, differed in several ways from our set-up. The previously published results were observed under conditions of more severe colitis induced by higher DSS concentrations [17,18], longer DSS treatment or by combinations with the carcinogen azoxymethane (AOM), which itself impairs the intestinal barrier and worsens colitis [17,21]. In those studies using 3 and 4% DSS, mortality rates were used as the main readout to describe the protective selenium effects. Whether this approach is suitable as a model for human IBD is questionable, as IBD patients also do not die from acute inflammation. Based on that, it appears that selenium only acts in an anti-inflammatory manner under conditions of severe inflammation. However, this hypothesis needs to be verified in an experiment aiming to directly compare selenium effects in mice treated with different DSS concentrations.

Another important difference between the studies was the selenium content of the diets used. The selenium-deficient diets of two previous studies contained less than 0.01 mg/kg diet [17,18], while we aimed to study the effects of a suboptimal supply with about half of the recommended intake for mice (0.07 mg/kg diet). We chose this selenium concentration to imitate the current selenium intake of the European population [16]. The absence of a pro-inflammatory effect indicates that only a manifest selenium deficiency worsens colitis symptoms, while even a suboptimal selenium supply appears to be enough to protect against colitis. Indeed, it has been shown recently that the selenoprotein expression of macrophages is essential for the colitis-suppressing effects of selenium. In mice with a macrophage-specific knockout of Trsp, the gene encoding for the selenoprotein-specific tRNA, and, thus, totally impaired selenoprotein expression, the selenium supply did not have any effect on colitis severity while effects were observed in wild-type controls [18]. We observed herein that the selenium-sensitive selenoprotein SelH was fully expressed in intraepithelial T-cells of lymphoid follicles despite a suboptimal selenium supply (Figure 7G). Previously, it has been shown that splenic leukocytes rank high in the tissue hierarchy and are still relatively well supplied with selenium, even though the intake was limited [12]. Consequently, only a severe selenium deficiency appears to affect selenoprotein expression in immune cells and, thereby, aggravates colitis. This raises the question of whether IBD patients with a suboptimal selenium status would profit from selenium supplementation and, if yes, under which exact circumstances.

We could herein show for the first time that a short-term selenite supplementation of DSS-treated mice during acute inflammation exacerbated colitis. The selenite dosage of 0.6 mg/kg diet, which is about four-times the recommended murine intake [28], was chosen to resemble human intervention studies with a daily supplementation of 200 μg [42,43], which is also about four-times the recommended human intake (55 μg per day) [44]. Different colitis parameters were increased in the short-term selenite group in comparison to the other experimental groups. Short-term selenite supplementation led to an elevated over-all inflammation score (Figure 3A), which includes parameters, such as weight loss (Figure 3B), duration and manifestation of diarrhea, presence of blood in the feces, swelling and shortening (Figure 3C) of the colon and different microscopically-evaluated characteristics of colon tissue damage. Furthermore, transcript levels of the pro-inflammatory cytokines Tnfα and Ifnγ, as well as Cox2 (Figure 4) were upregulated in the colon by short-term selenite supplementation. Plasma levels of the Th1-cytokines TNFα and IFNγ were increased, while the Th2-cytokines IL-5 and IL-6 were unaffected (Figure 5).

To identify putative mechanisms for the short-term selenite effect, we first characterized selenoprotein expression both in the liver and in the colon. Obviously, the systemic selenium supply was not completely replete after one week of selenite supplementation, indicated by significant differences in hepatic GPx activity between long- and short-term supplementation (Figure 6A). Also, compared to short-term SeMet, short-term selenite feeding caused a significantly lower GPx activity. This might be explained by the higher absorption efficiency of SeMet in comparison to selenite [45]. Less sensitive markers than hepatic GPx activity, like hepatic TrxR activity (Figure 6B) and Sepp1 plasma levels (Figure 6C), were already maximized after the short-term interventions. In the liver, Sepp1 expression is first optimized in order to transfer selenium to peripheral organs before GPx1 expression is increased [37].

In contrast to the liver, selenium status was fully maximized in the colon of short-term treated groups, indicated by GPx activity (Figure 7A) and transcript levels of the highly sensitive selenoproteins Selh (Figure 7C), Selw1 (Figure 7D) and Gpx1 (Figure 7E) [46]. Thus, the enhancement of colitis by short-term selenite supplementation obviously was independent from changes in selenoprotein expression. The only exception was the mRNA expression of Sepp1, which was upregulated upon short-term selenite supplementation compared to the suboptimal and short-term SeMet group (Figure 7F). This expression pattern resembles that of the analyzed inflammation markers and might rather reflect the increased amount of inflammatory cells in the sample than a higher expression in epithelial cells. Sepp1 is known to be expressed in different immune cells, like macrophages and T-cells [13,47]. GPx1, however, is also highly expressed in these immune cells and would therefore be expected to be upregulated accordingly. Furthermore, reporter gene assays of the Sepp1 promoter revealed that it is inhibited by pro-inflammatory cytokines in CaCo2 cells [48]. In a mouse model of LPS-induced sepsis, hepatic Sepp1 expression was only half the amount of untreated controls [49]. During the course of DSS-induced colitis, Sepp1 expression was downregulated in the colon with increasing disease severity [50]. In the current experiment, all mice received DSS, which makes it impossible to compare Sepp1 expression levels between treated and untreated mice. However, based on the published results, it would have been logical if Sepp1 expression levels were lowest in the group with the most severe inflammation, which is obviously not the case. Thus, the short-term selenite treatment itself enhanced Sepp1 expression in the colon via a yet to be elucidated mechanism. Further studies are needed to clarify the role of colonic Sepp1 expression during colitis development and whether this upregulation is necessary to modulate the short-term selenite effect.

Besides enhancing selenoprotein expression, high selenite concentrations are supposed to shift the redox balance of cells to a more oxidized state [51]. For example, in murine keratinocytes (MK-2), a 24-h treatment with selenite (5 μg/mL) resulted in increased levels of 8-OHdG, a marker for oxidative DNA damage [40]. However, these high concentrations were most probably not achieved in the colon of our selenite-supplemented mice. Analysis of 8-OHdG by immunohistochemistry of the colon revealed high levels in inflammatory cells, but only a weak staining in epithelial cells (Figure 8C,D). Scoring of the staining intensity in epithelial cells did not provide any difference between the experimental groups (Figure 8B). In addition, we analyzed NQO1 activity as a prototypical target gene of the redox-sensitive transcription factor Nrf2 [52]. In accordance with 8-OHdG, we did not observe any difference (Figure 8A). Nrf2 target genes are part of the adaptive response system, which is induced quickly to deal with any kind of stress. Thus, it might well be that the intestinal cells already adapted themselves to the higher selenite supply after seven days of treatment. For assessing the oxidative effects of selenite, it might be more conclusive to analyze animals directly one day after the diet switch from a suboptimal to a selenite supplemented one. Acute selenite feeding of selenium-deprived animals might also result in selenite accumulation due to the low expression level of the selenium-dependent Trx/TrxR system (Figure 7B), which is mainly responsible for metabolizing selenite [26]. If selenite cannot be reduced by the thioredoxin or glutaredoxin systems, it can also react with GSH to form GSSeSG. A knockdown of TrxR1 in malignant mouse DT cells or NIH3T3 mouse embryonic fibroblasts resulted in the formation of GSSeSG and thus enhanced selenite toxicity [53]. GSSeSG is supposed to induce apoptosis independent of changes in the cellular redox status [54]. Interestingly, inorganic iron supplementation of DSS-treated rats has also been shown to increase intestinal inflammation, which is supposed to be a result of higher superoxide levels due to the iron-mediated Fenton reaction [55,56]. However, it is also well established that, e.g., H_2_O_2_ is an important signaling molecule essential for the optimal function of innate and adaptive immunity [57] and thus, any modification of the cellular redox status has the potential to interfere with the immune response. Further studies are needed to elucidate the exact mechanisms of short-term selenite supplementation during acute colitis.

## 5. Conclusions

In contrast with earlier publications, suboptimal selenium status did not aggravate inflammation using a moderate DSS-induced colitis model. However, a short-term selenite supplementation during the acute colitis exacerbated colitis severity. In order to provide recommendations for patients with inflammatory bowel disease, it would be helpful to follow-up on these findings using clinical studies.
